# Processing of *OPA1* with a novel N-terminal mutation in patients with autosomal dominant optic atrophy: Escape from nonsense-mediated decay

**DOI:** 10.1371/journal.pone.0183866

**Published:** 2017-08-25

**Authors:** Aneta Ścieżyńska, Ewelina Ruszkowska, Kamil Szulborski, Katarzyna Rydz, Joanna Wierzbowska, Joanna Kosińska, Marek Rękas, Rafał Płoski, Jacek Paweł Szaflik, Monika Ołdak

**Affiliations:** 1 Department of Histology and Embryology, Medical University of Warsaw, Warsaw, Poland; 2 Department of Ophthalmology, Medical University of Warsaw, Warsaw, Poland; 3 Department of Ophthalmology, Military Institute of Medicine, Warsaw, Poland; 4 Department of Medical Genetics, Medical University of Warsaw, Warsaw, Poland; Medizinische Universitat Innsbruck Department fur Kinder- und Jugendheilkunde, AUSTRIA

## Abstract

Autosomal Dominant Optic Atrophy (ADOA) is the most common dominantly inherited optic neuropathy. In the majority of patients it is caused by *OPA1* mutations and those predicted to introduce a premature termination codon (PTC) are frequently detected. Transcripts containing PTC may be degraded by nonsense-mediated mRNA decay (NMD), however very little is known about an effect of *OPA1* mutations on NMD activation. Here, using a combination of linkage analysis and DNA sequencing, we have identified a novel c.91C>T *OPA1* mutation with a putative premature stop codon (Q31*), which segregated with ADOA in two Polish families. At the mRNA level we found no changes in the amount of *OPA1* transcript among mutation carriers vs. non-carriers. Specific allele quantification revealed a considerable level of the *OPA1* mutant transcript. Our study identifies a novel pathogenic *OPA1* mutation and shows that it is located in the transcript region not prone for NMD activation. The data emphasizes the importance of analyzing how mutated genes are being processed in the cell. This gives an insight into the molecular mechanism of a genetic disease and promotes development of innovative therapeutic approaches.

## Introduction

Autosomal Dominant Optic Atrophy (ADOA; OMIM: #165500) is the most common form of inherited optic neuropathy leading to progressive bilateral reduction of visual acuity, centrocecal scotomas of variable density, color vision deficits and temporal optic disc pallor, which begin in early childhood [1, 2]. Due to variable inter- and intrafamiliar expressivity of ADOA visual impairment may range from mild to severe [3–5]. In about 10–20% of ADOA cases a more severe ADOA “plus” phenotype (DOA+; OMIM: # 125250) with extra-ophthalmic manifestation e.g. sensorineural hearing impairment and neuromuscular symptoms has been reported [6–8].

To date, five loci have been linked to ADOA but in about 60–80% of cases mutations in the *OPA1* gene (OMIM: *605290) are identified. The majority of them are nonsense, frameshift or splice site mutations, which are predicted to introduce a premature termination codons (PTCs) [9]. Transcripts containing PTC may undergo degradation in the process of nonsense mediated mRNA decay (NMD) protecting cells from a dominant negative or gain of function effects. NMD prevents accumulation of truncated peptides, even though some of these proteins could be fully or partially functional [10–12].

Currently there is no effective treatment for ADOA, however, new promising technologies aimed at NMD suppression and PTC read-through are tested in different genetic diseases. This approach may restore the capacity of a cell to synthesize a full-length protein from a mutated transcript. Implementation of the therapies requires knowledge on the consequences a mutation may exert on gene processing. Low abundant mRNAs due to transcript degradation by NMD may constrain the read-through therapies [13]. This clearly indicates the importance of personalized studies for the analysis of molecular mechanism of genetic diseases and possible treatment options.

As of writing the manuscript only few studies have addressed the important aspects of the fate of mutated *OPA1* transcripts. Here we present a novel *OPA1* p.Q31* mutation and processing of the mutated *OPA1* gene at the mRNA level in two families with ADOA.

## Materials and methods

### Patients and clinical investigation

Two families A (n = 11) and B (n = 6) were included in the study. Written informed consent was obtained from all participants. All clinical investigations have been conducted according to the principles expressed in the Declaration of Helsinki. The study was approved by the Ethics Committee at the Medical University of Warsaw (KB/191/2010). Detailed ophthalmological examinations with electrophysiological testing (pattern visual evoked responses, full-field electroretinography) were performed with use of RETIscan/RETIport system (Roland Consult, Wiesbaden, Germany). Prior results of audiological and neurological assessment were uneventful.

### Linkage analysis and *OPA1* gene sequencing

DNA was extracted from blood by a standard salting out method. For amplification of microsatellite markers (D3S3642, D3S3590, D3S2305, D3S3562, D3S2748) PCR primer sequences were retrieved from the Ensembl Genome Browser (http://www.ensembl.org). Forward primers were 5’labeled with 6-carboxyfluorescein (6-FAM) and reverse primers were unlabeled. PCR products were mixed with a fluorescent size marker (Applied Biosystems, Foster City, CA, USA) and analyzed on ABI PRISM 377 DNA sequencer (Applied Biosystems). Fragment lengths were analyzed with the Gene Mapper software (Applied Biosystems).

For the amplification of all *OPA1* exons PCR primers were designed with the Primer 3 Plus public domain software (http://primer3plus.com/cgi-bin/dev/primer3plus.cgi) and the *OPA1* gene NG_011605.1 reference sequence. The *OPA1* exon amplicons were sequenced using the BigDye Termination cycle sequencing kit v3.1 (Applied Biosystems) and analyzed on ABI PRISM 377 DNA sequencer (Applied Biosystems). The results were visualized by the Variant Reporter Software v1.1 (Applied Biosystems). PCR primers and reaction conditions are available upon request.

### RNA isolation, cDNA synthesis and quantitative mRNA measurements

Peripheral blood mononuclear cells (PBMC) were isolated using Gradisol L (d = 1077 g/cm^3^, Aqua-Med, Lodz, Poland) according to a standard procedure. Total RNA was extracted from the cells with RNeasy Mini Kit (Qiagen, Hilden, Germany). For reverse transcription 0.5 μg RNA and QuantiTect Reverse Transcription kit (Qiagen) were used. Quantitative PCR (qPCR) was performed on 7500 Real-Time PCR System (Applied Biosystems) with probes from the Roche Universal Probe Library (Roche, Basel, Switzerland) and PCR primers (Oligo IBB PAN, Warsaw, Poland) designed using the Probe Finder software (Roche). For human *OPA1* qPCR primer pair 5’-TCAAGAAAAACTTGATGCTTTCA-3’ (exon 29) and 5’-GCAGAGCTGATTATGAGTACGATT-3’ (exon 30) and probe no.2 (exon 29–30) were used. *GAPDH* was amplified using primer pair 5’-CCCCGGTTTCTATAAATTGAGC-3’ and 5’-CACCTTCCCCATGGTGTCT-3’ and probe no.63. Expression values for *OPA1* were normalized to *GAPDH* and quantified with the Pfaffl method [14]. All samples were run in triplicates.

Expression of *OPA1* from the mutated allele was measured using a modified PCR-RFLP method. *OPA1* exon 2 was PCR amplified from cDNA with 5’-GCTCTGGAATAAAAGGAAGTTTACCAT-3’ forward and FAM-conjugated 5’-GCGAAGTTTTAAGAGTCTCGTAGC-3’ reverse primers to introduce a C>T mismatch (underlined), which together with the *OPA1* c.91C>T (p.Q31*) mutation created an AanI restriction site. Digestion with AanI (Thermo Scientific, Waltham, MA, USA) in the presence of the heterozygous c.91C>T mutation resulted in 29, 213 and 242 bp products, in the wild-type homozygotes only the 242 bp product was observed. Digestion products were analyzed on the ABI PRISM 377 DNA sequencer (Applied Biosystems) and peak areas were quantified with GeneMarker (SoftGenetics LLC, State College, PA, USA). The log-transformed ratios between the wild-type and mutated transcripts peak areas were converted to relative amounts of transcripts by their extrapolation from a regression line [15].

### Statistical methods

Statistical differences between the groups were analyzed with a two-sided unpaired t-test from GraphPad Prism 5 (GraphPad Software, La Jolla, USA). P values <0.05 were considered as significant.

## Results

### Clinical features of optic atrophy in the studied families

**Fig 1:**
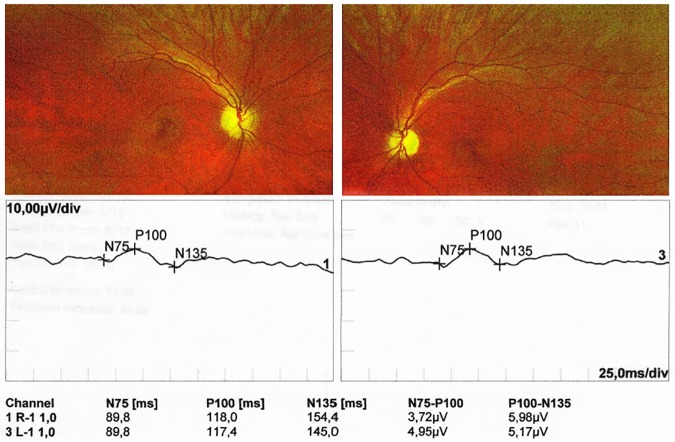
Characteristic features on ophthalmological evaluation in the proband from family A. Fundus examination showed bilateral temporal pallor of the optic disc. PVEP presented a reduced amplitude and delayed latency of P100 wave. Components of PVEP, N75, P100 and N135, indicate polarity (negative or positive) and absolute latencies (msec) of the peaks waveforms. Right panel—left eye, left panel—right eye.

The proband from family A (PatID 217) had vision deterioration from the age of 6 years. Ophthalmological examination at the age of 28 years revealed temporal pallor of the optic nerve disc and color vision impairment in the red-green axis in both eyes. The best corrected visual acuity (BCVA) on Snellen charts was 0.4 and 0.5 in right and left eye, respectively. The pupillary response to light was abnormal. Visual field examination showed non-characteristic scotomas in the upper hemispheres in both eyes. Pattern visual evoked potentials (PVEP) presented delayed P100 wave latency (Fig 1). The full-field electroretinography (F-ERG) was within the normal limits, showing the normal function of retina.

The proband from family B (PatID 239) noticed a reduced visual acuity in the first decade of life. Eye examination at the age of 32 showed temporal pallor of the optic nerve disc in both eyes and impairment in color vision and pupillary responses. BCVA was 0.5 in both eyes. Visual field examination showed non-characteristic peripheral scotomas in both eyes. PVEP showed delayed P100 wave latency (133 ms in the right and 119 ms in the left eye) with a slight decrease of the P100 wave amplitude. The full-field electroretinography (F-ERG) was within the normal limits.

**Fig 2:**
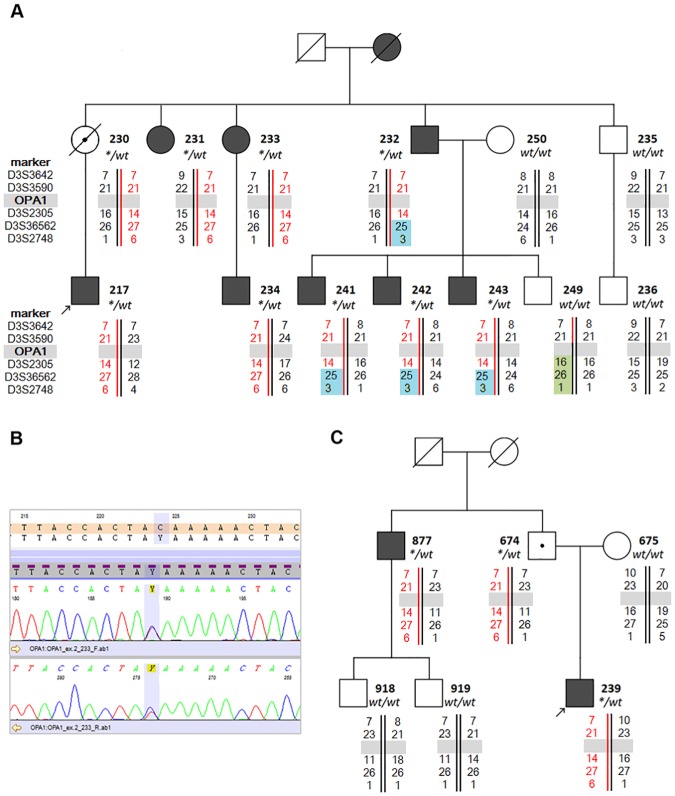
Identification of a novel p.Q31* *OPA1* mutation. Pedigrees of the investigated ADOA family A (A) and B (C). Probands are indicated by arrows. Black symbols denote affected subjects, white symbols denote unaffected subjects without *OPA1* mutation. Symbols with a black dot inside refer to healthy mutation carriers. Bolded numbers next to the symbols denote the respective DNA samples (AC). Marker names (analyzed for both families) are shown on the left of the family A pedigree. Unbolded numbers to the right of the marker names denote microsatellite repeat motif sizes. The grey rectangle marks the location of the *OPA1* gene within the 3q28–29 region (AC). Wt/wt refers to patients homozygous for the wild-type allele, while */wt to patients heterozygous for the p.Q31* *OPA1* mutation (AC). Electropherogram from Sanger bidirectional sequencing of the *OPA1* exon 2 shows the c.91C>T (CAA>UAA) heterozygous mutation, predicting a premature stop codon p.Q31* in the OPA1 protein (B).

The phenotypes of both probands were consistent with the clinical features of optic atrophy. Except for the probands, we have identified seven affected family members in family A and one in family B. Pedigree analysis in both families indicated that optic atrophy was inherited in an autosomal dominant mode with incomplete penetrance (both probands had unaffected parents) (Fig 2A and 2C). Affected individuals did not present any other additional symptoms.

### Microsatellite markers encompassing the *OPA1* gene segregate with optic atrophy

Microsatellite study performed for family A and B (Fig 2A and 2C) revealed segregation to *OPA1*, the major optic atrophy locus. Study of the distribution of microsatellite markers surrounding the *OPA1* gene (3q28–29) revealed that PatID 232 has inherited from his mother chromosome 3, which most probably underwent a crossing-over event encompassing the D3S3562 marker but not the *OPA1* gene. The patients and three of his sons who received the chromosome 3 were affected. His fourth son (PatID 249) was unaffected. He inherited the same chromosome 3, which underwent another recombination event swapping more a proximal region of chromosome 3q (D3S2305 and *OPA1*). These data provided another indication that *OPA1* is involved in the development of optic atrophy in the family. The family A and B share the same size of microsatellite markers encompassing the mutated *OPA1* gene. Presence of the same haplotype within the 3q region of interest shows that both families may be considered as one kindred.

### Identification of a novel p.Q31* mutation in the *OPA1* gene

In both probands sequencing of all *OPA1* coding regions revealed the presence of a novel, heterozygous transition c.91C>T (p.Q31*) in exon 2 (Fig 2B). The mutation affects a highly conserved amino acid residue and introduces a premature termination codon into the OPA1 protein. Further analysis unveiled segregation of p.Q31* with optic atrophy in both families. Proband’s mother from family A and proband’s father from family B were unaffected carriers of p.Q31* (Fig 2A and 2C). In our study the proportion of individuals carrying the p.Q31* and expressing the disease phenotype was 10 out of 12, which constitutes a high penetrance of the mutation (83%). The p.Q31* mutation was not found in 1566 subjects from the Polish population (R Ploski, unpublished data).

### Considerable amount of *OPA1* mutant transcripts in p.Q31* carriers

**Fig 3:**
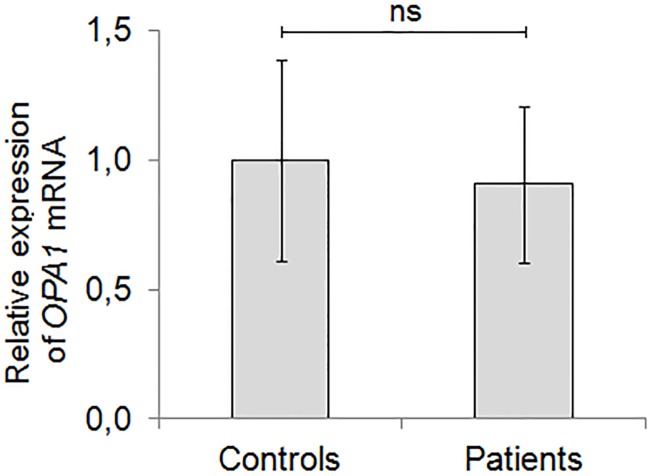
*OPA1* expression at mRNA level. *OPA1* mRNA level were quantified in PBMC in patients with p.Q31* mutation and control samples from healthy individuals. The amount of *OPA1* mRNA was quantified by real-time PCR in relation to *GAPDH*. The values of control samples were set at 1.

Next, we evaluated the consequences of the p.Q31* mutation on the *OPA1* gene mRNA level in PBMC. No statistically significant differences were observed in the amount of *OPA1* transcripts measured in p.Q31* mutation carriers (n = 5) and wild-type controls (n = 7) (p = 0.423) (Fig 3). Presence of the mutant *OPA1* transcripts was confirmed in all investigated patients with the p.Q31* mutation (n = 5). The mutant transcripts constituted about 47% of the wild type *OPA1* transcripts as assessed by allele-specific transcript quantitation (Table 1).

**Table 1:** Quantification of the *OPA1* mutant transcripts.

PatID	$\frac{Wild\;type\;peak\;area}{Mutant\;peak\;area}$	$Log\left(\frac{Wild\;type\;peak\;area}{Mutant\;peak\;area}\right)$	% mutant of wild type allele
**217**	2.08	0.32	50.14
**231**	2.06	0.31	48.95
**239**	2.34	0.37	42.43
**242**	2.08	0.32	47.76
**674**	2.09	0.32	47.12
**Mean values**	2.13	0.33	47.28

Log-transformed ratios were extrapolated from a standard curve to absolute figures (according to [15]).

## Discussion

The majority of *OPA1* mutations have been identified in its 3’ region leaving by far less mutations in the first five exons [9], [16]. In this study we have identified a novel nonsense mutation p.Q31* in the second exon of the *OPA1* gene in all affected family members from two families and show that it exhibits a high penetrance (83%). *OPA1* mutations in ADOA patients display a penetrance from 43% to 100% [8]. High penetrance of the p.Q31* may be explained by its deteriorating effect on the protein structure and function. The premature stop codon is presumed to terminate translation just after 30 amino acids, which leads to a dramatic reduction of almost 97% of the OPA1 protein length.

Different stop codons have different read-through capacities inversely correlated with their termination fidelities. The UGA stop codon has the highest read-through potential, while UAA the lowest. *OPA1* p.Q31* mutation introduces a UAA stop codon supposedly with a high termination fidelity but it should be taken into account that the read-through capacity of a stop codon may be directly altered by adjacent sequences and/or indirectly by cell- and tissue-specific changes affecting the NMD activity and subsequently mRNA levels from which functional protein might be produced [11]. Thus, each stop codon should be individually examined for a potential therapeutic outcome of read-through compounds. Currently, for patients bearing nonsense mutations novel therapeutic approaches are being tested. Chemical compounds e.g. aminoglycosides or ataluren induce a read-through of the premature termination codon (PTC) during protein translation. PTC124 has been recently approved in the European Union for the treatment of Duchenne muscular dystrophy caused by a nonsense mutation. Patients with higher amounts of mutated transcripts respond better to this therapy [17].

In our patients we did not detect significant differences between the total amounts of *OPA1* mRNA as compared to control subjects. It strongly suggested that the pool of *OPA1* mRNA in the patients contains the mutant transcripts and NMD is weak or absent. In the presence of NMD the amount of mutant mRNA level is considered to be reduced to 10–40% expression level of the wild type transcripts [18] or even higher, i.e. to ~5–25% [19]. For *HNF-1α* mutant transcripts NMD is thought to occur if the PTC-containing transcripts constitute <0.01–40% of the transcripts from normal alleles [20], while for the *FBN1* gene the numbers are even reduced to 2–28% levels of the wild type transcript [21].

We quantified the levels of p.Q31* mutated and wild-type *OPA1* transcripts and found that the *OPA1* mutant transcript is not prone for NMD degradation. This may be explained by a higher resistance for NMD-driven degradation due to a short open reading frame of the mutant transcripts [12]. It was noted that length of upstream ORF (uORF) may trigger NMD and in transcripts with short uORF NMD is inhibited independently from the sequence context [22, 23]. In plants the length of uORF that might activate NMD was at least 35 codons [24] but as of writing the manuscript it has not been determined for human cells. Our data are consistent with the results of Schimpf et al., who found that two *OPA1* mutations i.e. p.W2* and p.R52* surrounding p.Q31, did not activate NMD [12]. According to our knowledge no study other than that of Schimpf et al. investigated the susceptibility of *OPA1* mutant transcripts to NMD. Our study, for the first time, provides the data for the novel p.Q31* *OPA1* mutation and an independent confirmation that the NMD incompetent area encompasses the second exon of the *OPA1* gene.

Our study presents novel p.Q31* *OPA1* mutation identified in two one kindred families with ADOA. We present unchanged level of mutated *OPA1* transcripts. This mutation adds to exceptions not prone for the NMD activation process. Our data highlights the important role of personalized mutation analysis precluding treatment approaches focused on NMD silencing and read-through therapies.
